# Interaction between Six Waxy Components in Summer Black Grapes (*Vitis vinifera*) and Mancozeb and Its Effect on the Residue of Mancozeb

**DOI:** 10.3390/ijms24097705

**Published:** 2023-04-22

**Authors:** Boru Guo, Aying Wen, Hang Yu, Yahui Guo, Yuliang Cheng, Yunfei Xie, He Qian, Weirong Yao

**Affiliations:** 1 State Key Laboratory of Food Science and Technology, Jiangnan University, Wuxi 214122, China; 2 School of Food Science and Technology, Jiangnan University, Wuxi 214122, China; 3 Collaborative Innovation Center of Food Safety and Quality Control in Jiangsu Province, Jiangnan University, Wuxi 214122, China

**Keywords:** mancozeb, wax of the grapes, pesticide combination, molecular docking, IGMH

## Abstract

Mancozeb, an antifungal typically used for the growth of fruits, has the characteristic of non-internal absorption, and has a risk of binding to the waxy components of fruits. This work investigated the interaction of pesticide molecules with the waxy layer on the grape surface and their effects on pesticide residues in grapes. The study observed significant changes in the compositions of the waxy layer on the grape surface after soaking in a mancozeb standard solution. The six substances—oleanolic acid, ursolic acid, lupeol, octacosanol, hexacosanal, and γ-sitosterol—with discernible content differences were chosen for molecular docking. Docking results were further visualized by an independent gradient model based on Hirshfeld partition (IGMH). Hydrogen bonds and van der Waals forces were found between mancozeb and the six waxy components. Moreover, the negative matrix effects caused by the presence or absence of wax for the determination of mancozeb were different through the QuEChERS-HPLC-MS method. Compared with the residue of mancozeb in grapes (5.97 mg/kg), the deposition of mancozeb in grapes after dewaxing was significantly lower (1.12 mg/kg), which further supports that mancozeb may interact with the wax layer compositions. This work not only provides insights into the study of the interaction between pesticides and small molecules but also provides theoretical guidelines for the investigation of the removal of pesticide residues on the surface of fruits.

## 1. Introduction

The grapes have a long history of cultivation and are grown in a wide range of areas. Its fruit can be used as fresh or preserved fruit and also to make wine. At the same time, due to its rich phenolic substances, it is also used as an essential raw material for extracting plant antioxidants [1]. Therefore, grapes are regarded as an important economic crop worldwide. The surface of grapes is often covered with a layer of “hoarfrost”-like substances, which is the waxy surface layer of grapes, mainly including long-chain aldehydes, primary alcohols, alkyl esters, alkanes, and triterpenoids [2]. The pentacyclic triterpenoids, such as oleanolic acid and its ursolic isomer acid, have anti-cancer, anti-inflammatory, anti-diabetic, and anti-multiple sclerosis effects [3]. Lupeol, one of the tetracyclic triterpenoids, also has significant anti-inflammatory and anti-cancer effects [4]. In addition to health benefits, these triterpenoids are also involved in the structure of the waxy layer [5]. A previous study has shown a molecular model of the spatial arrangement of oleanolic acid and the major aliphatic compound n-hexadecanol to explain the organization of the grape cuticle. Oleanolic acid can form dimers through hydrogen bonding interactions between the hydroxyl group of one molecule and the carboxyl group of a second molecule. The remaining functional groups may interact with complementary groups of other molecules, including fatty alcohols, to form three-dimensional arrangements [5]. Due to the covering of these waxy components, the surface of the grapes presents a hydrophobic feature which can protect the internal water of the grapes from loss [6]. It also reduces the infestation of some pests and diseases [7] and enhances the mechanical strength of grape peel [8]. However, some studies have proposed that the waxy layer structure on the surface of fruits may provide a way for hydrophobic pesticides to penetrate plant tissues [9,10].

Mancozeb, as an ethylenebis(dithiocarbamate)s (EBDCs) pesticide, is a coordination compound of manganese ethylene dithiocarbamate and zinc ions [11]. It has a bactericidal effect by inhibiting the oxidation of pyruvic acid in bacteria, and the manganese and zinc elements it contains significantly strengthen fruits [12]. Due to its high efficiency and low toxicity [13], mancozeb is often used to control various diseases, such as anthracnose and early fruit blight. Among the fungicides with one application frequency in a growing season, 33.78% of the growers choose mancozeb, ranking first [14]. Mancozeb is insoluble in water and most organic solvents and easily remains on the surface of fruits. It is unstable when exposed to light, heat, and humidity, and quickly degrades into ethylene thiourea (ETU) and other products. ETU poses a risk to human health. It can be teratogenic, carcinogenic, or mutagenic, and long-term exposure may lead to the risk of thyroid cancer [15,16,17,18,19].

At present, different kinds of literature have paid attention to the effect of epidermal wax on the removal or degradation of pesticides. Most of them think that the components in the wax layer have chemical or physical interactions with the pesticides, which affects the degradation effect of pesticides. Several studies have shown that epidermal wax affects the photodegradation of pesticides [20,21,22,23,24,25]. When the types of epidermal waxes contacted by pesticides differ, the types and contents of photodegradation products are also quite different. In addition, the presence of a waxy layer affects the efficiency of pesticide removal from the surface of fruits. For smooth lettuce containing more surface wax, the mancozeb appears to be more easily removed, which the authors explained was because it readily migrates from its waxy surface to water [26]. Long-chain esters and alkanes that accumulate in apple leaves during the growing season also cause a rapid decrease in pesticide residues from foliar sprays of mancozeb [27]. These seem to indicate that the presence of wax is less likely to produce pesticide residues. However, some experiments yield the opposite results. For example, the concentration of azoxystrobin in washed apples was lower in samples with wax removed than in samples without wax extracted from the cuticle [9]. Pesticide residues on citrus and waxed apples are primarily concentrated in the epidermal wax [28,29], which is thought to be because those pesticides may dry on the surface or bind to the wax outside the peel and then adhere to the peel, thus remaining non-removable in the wash treatment [30]. However, the interaction between mancozeb and waxy layer components has not been studied in-depth, and there is a lack of explanation at the molecular level.

Intermolecular interactions are thought to be a possible cause of pesticide enrichment in waxes. Molecular docking is a bioinformatics-based theoretical modelling technique that examines the interactions of molecules (such as ligands and receptors) and makes computer-based predictions about their binding affinities and modes [31]. Several studies have cited the AutoDock semi-flexible docking software, which consists of two components: AutoGrid and AutoDock. AutoDock is primarily utilized as a search tool to identify the ideal conformation and score, while AutoGrid is used to calculate the energy level contained in the lattice. However, it cannot intuitively display the range and magnitude of weak intermolecular interactions, so it needs to be combined with another visualization method. Multiwfn [32] is a wave function analysis software developed by Lu Tian et al. Multiwfn users have widely used the independent gradient model (IGM) to display weak interactions between molecules [33] graphically, and the independent gradient model based on the Hirshfeld partition (IGMH) is the latest upgraded version of IGM. As a result, the isosurface created by the IGMH approach has replaced the IGM method as the suggested visualization technique for weak intermolecular interactions.

This study was performed on summer black grapes with a thick wax layer. First, the changes in wax components before and after soaking pesticides were analyzed by GC-MS. Following that, the interactions between pesticide molecules and wax components were simulated through the molecular docking method and then visualized with Multiwfn. Finally, the residue of mancozeb in grapes before and after dewaxing was determined by the QuEChERS-HPLC-MS method to verify the association of mancozeb and wax components. The work provides theoretical guidance for removing pesticides on the surface of fruits.

## 2. Results and Discussion

### 2.1. Effect of Solvent Extraction on the Wax Layer

**Figure 1:**
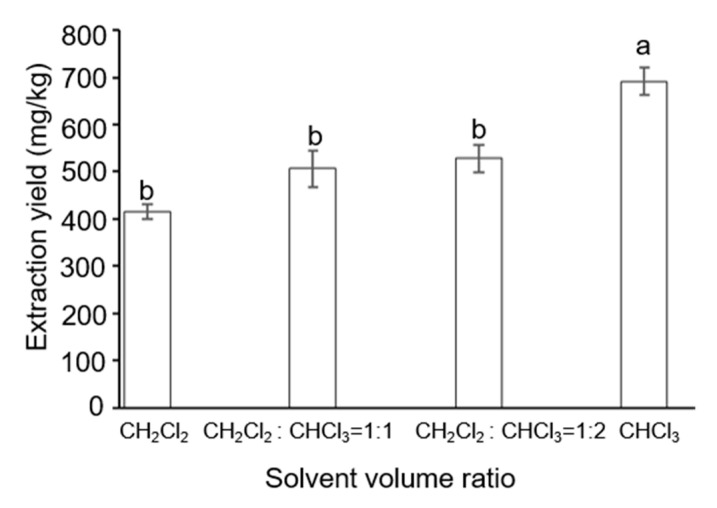
The extraction amount of grape wax under different solvents. a and b represent significant differences between the data, and the differences between the data corresponding to different letters are significant (*p* < 0.05).

Epidermal wax of plants is made up of a complex mixture of diverse substances—mostly aliphatic acid, alkane, aliphatic alcohol, aldehyde, ketone, and ester. Furthermore, different plant species’ epidermal wax components may vary [33]. The solvent is a crucial factor affecting the extraction effect of grape wax. The result of solvent extraction is often related to the wax composition of the grape epidermis, following the principle of similar phase solubility. When extracting, a solvent with high wax yield and good volatility should be selected, which is convenient for the later collection of wax in the solvent. Based on this, dichloromethane and chloroform were selected as extraction solvents to study the effect of wax extraction under different solvent ratios. The experimental results are shown in Figure 1.

Solvents of intermediate polarity should maximize the solubility of all wax constituents, including the highly hydrophobic hydrocarbons and the much more polar compounds containing (many) functional groups [34]. Figure 1 shows a significant correlation between the proportion of chloroform and the extraction amount (*p* < 0.05). The higher the content of chloroform, the greater the extraction amount, which is consistent with previous research findings [35]. These experiments have shown that the waxy layer on the surface of grapes has the best extraction effect under the condition of pure chloroform. The epidermal wax of fresh and pesticide grapes was extracted using chloroform as the extraction solvent. This process was done ten times, and the statistics of the wax content are displayed in Table S1. There was no significant difference in the content of the waxy layer extracted from fresh and pesticide grapes. The results indicated that soaking pesticides would neither prevent chloroform from extracting the waxy layer on the surface nor improve the yield of the waxy layer.

**Figure 2:**
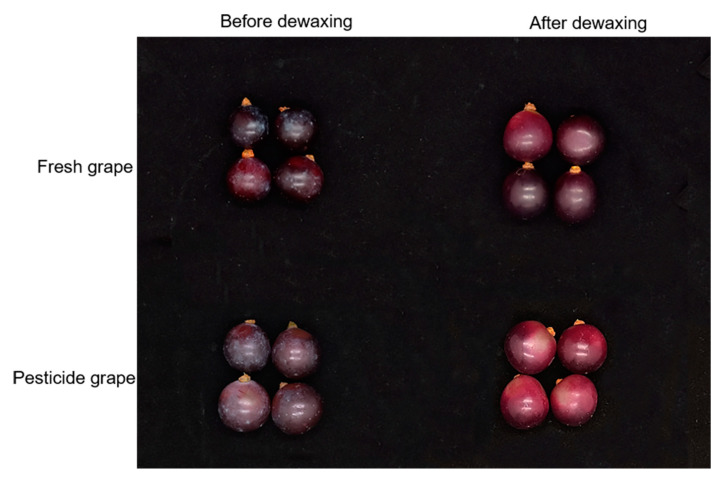
Comparison of fresh grapes and pesticide grapes before and after dewaxing.

**Figure 3:**
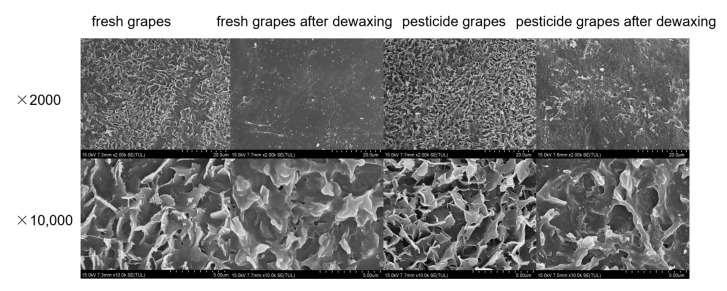
Electron microscope picture of grape peel.

The surface morphology of grapes before and after dewaxing is shown in Figure 2. Combined with the grape epidermis electron microscope results in Figure 3, the waxy layer structure on the surface of grapes is like platelets with irregular edges protruding vertically from the surface. The morphology of plant surface waxes is often related to its main components. For example, the waxy structure of primary alcohols is generally lobed or serrated; nonacosan-10-ol tubules or β-diketone tubules make waxes form tubular structures, etc. [36]. The main components of grape wax are primary alcohols, aldehydes, ketones, and triterpenoids with a particular chain length [37,38], corresponding to their sheet-like waxy layer structure, which is the same as the previous research result [7,39,40]. The surface wax of grapes was largely removed with chloroform. The lack of a clear difference between the morphology of surface wax before and after the grapes were soaked in pesticides suggests that simply soaking in the pesticides was insufficient to alter the wax layer’s composition. However, several studies have also demonstrated that applying pesticides to plants throughout their growth stage might change the wax layer’s makeup and build-up [41,42].

### 2.2. Waxy Layer Composition Analysis

**Figure 4:**
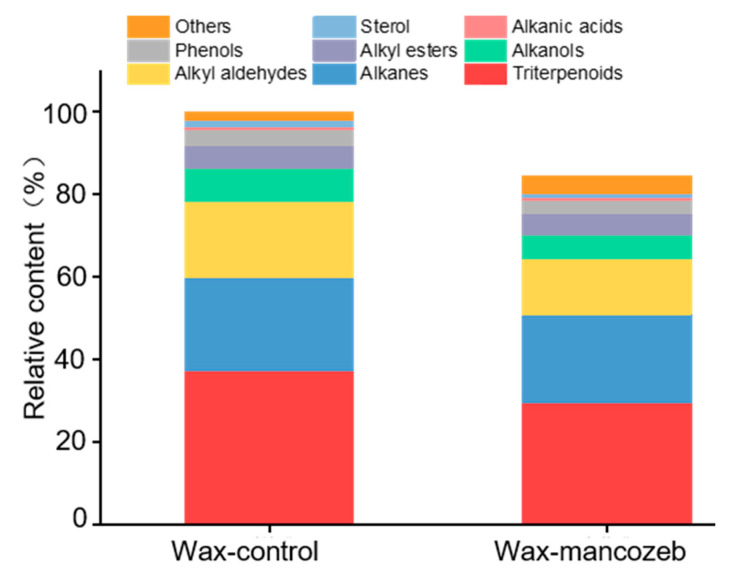
Statistics on the relative content of raw grape wax and pesticide grape wax components.

GC-MS analysis was conducted on grape wax-control and pesticide wax-mancozeb. The composition of grape wax is very complex, and direct detection can detect most long-chain alkanes, some alkanes, and a small portion of alkanes; Some components, such as oleanolic acid and ursolic acid, can only be detected after derivatization through BSTFA, because BSTFA reagent can convert compounds containing hydroxyl into corresponding trimethylsilyl derivatives, improving their volatility and thermal stability. In terms of composition, a total of 318 grape wax-control components were detected before derivatization, and 77 were detected after derivatization; 274 types of wax-mancozeb were detected before derivatization, and 60 types were detected after derivatization. Therefore, considering that the original grape wax content was 100%, the types of components detected in pesticide grape wax are only 84.5% of the original grape wax. The statistics of the relative content of components in the two types of wax are shown in Figure 4.

Overall, triterpenoids are the main components in the epidermal wax-control of *Vitis vinifera ‘Summer Black’* grapes, accounting for 37.15% of the original grape wax. They are followed by various long-chain alkanes (22.61%), alkyl aldehydes (18.69%), alkanols (8.13%), alkyl esters (5.36%), phenols (4.00%), and sterols (1.50%). In the wax-mancozeb, while there are fewer types of detected components, the relative amounts of the above types of substances also decrease. The decrease in sterols was the highest at 30.13%, followed by a decrease of 28.74% in alkanols, 28.10% in aldehydes, and 21.05% in triterpenoids. Perhaps a portion of the wax components that are easily soluble in water were washed away due to being soaked in pesticides, resulting in fewer types of detected components than the original grape wax. It is also possible that the presence of pesticides affects some structures of wax components, making them more difficult to detect, resulting in a decrease in relative content.

**Figure 5:**
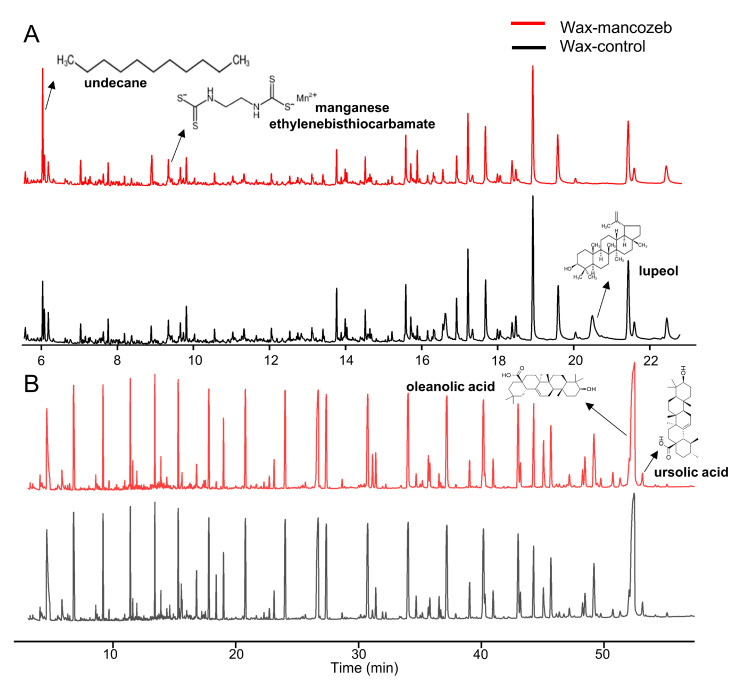
(**A**) Non-derivatized chromatograms and (**B**) derivatized chromatograms of the pesticide group and the control group.

The gas chromatograms of wax-control and wax-mancozeb for component detection are shown in Figure 5. In Figure 5A, only the wax-mancozeb group could detect pesticide fragments, namely [ethylenebis-(dithiocarbamate)]-manganese (C_4_H_6_MnN_2_S_4_). For the original wax components, most of them were seen in the wax-control group, such as lupeol (C_30_H_50_O), octacosanol (C_28_H_58_O), hexadecanoal (C_26_H_52_O), γ-sitosterol (C_29_H_50_O), and other substances. Oleanolic acid (C_30_H_48_O_3_) and ursolic acid (C_30_H_48_O_3_) need to be derivatized to be detected, and they are shown in Figure 5B. The peak area they noticed in the wax-mancozeb group was smaller than in the wax-control group, which may be due to the interaction of mancozeb with these substances, making the substances less captured by the TOF detector [43]. However, there are also cases where the peak value of the wax-mancozeb group is higher. For example, compared with the wax-control group, the detection response of undecane (C_11_H_24_) in the wax-mancozeb is increased, possibly due to the pesticide’s positive matrix effect. Another example is 2,2′-methylenebis(4-methyl-6-tert-butylphenol) (C_23_H_32_O_2_), ethyl tetracosanoate (C_26_H_52_O_2_), heptadecanoic acid ethyl ester (C_19_H_38_O_2_), and other substances. However, these substances are initially present in tiny amounts, and the difference between the two groups may be due to systematic errors in the instrumental analysis. More information for the above substances is provided in Table S2.

### 2.3. Molecular Docking Simulation and IGMH Analysis between Mancozeb and Waxy Components

**Figure 6:**
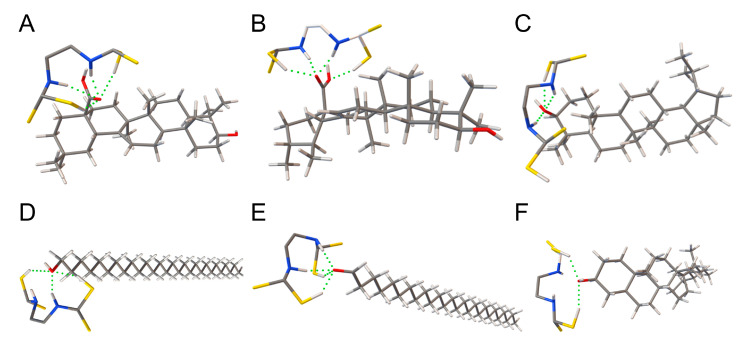
Molecular docking simulation of mancozeb and waxy components. (**A**) Oleanolic acid, (**B**) ursolic acid, (**C**) lupeol, (**D**) octacosanol, (**E**) hexacosanal, and (**F**) γ-sitosterol.

The substance molecules with high content in the wax layer and significant differences in the range (oleanolic acid, ursolic acid, lupeol, octacosanol, hexacosanal, and γ-sitosterol) between the pesticide group and the control group were selected for molecular docking simulation in Autodock. The hydrogen bonds formed by docking are shown in green dotted lines in Figure 6.

The docking results of oleanolic acid, ursolic acid, lupeol, octacosanol, hexadecanol, and γ-sitosterol and mancozeb in Autodock showed that the H on the imino group and the sulfhydryl group of the pesticide molecule can form a hydrogen bond between the O of the alcoholic hydroxyl group and the carboxyl hydroxyl group in the six wax components, and their minimum binding energies are −5.96 kcal/mol, −5.87 kcal/mol, −3.30 kcal/mol, −3.67 kcal/mol, −3.82 kcal/mol, and −2.22 kcal/mol. Generally, the docking results can be considered feasible when the lowest binding energy is less than −1.2 kcal/mol. The results indicate that the presence of the waxy layer binds to the pesticide molecules by forming hydrogen bonds. More detailed information on the docking results is given in Table S3.

**Figure 7:**
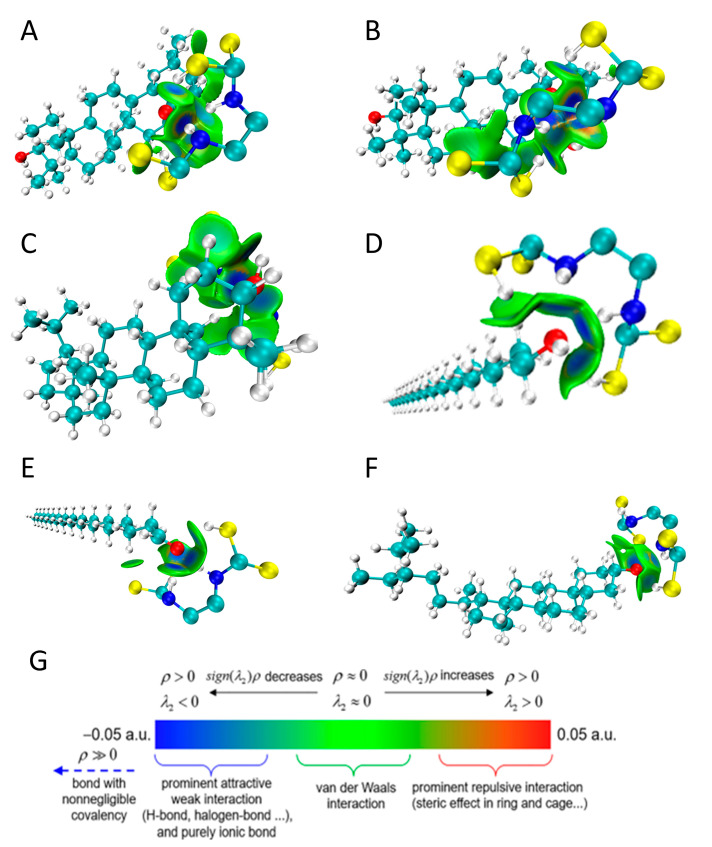
Isosurface of weak intermolecular interaction between mancozeb and waxy components by IGMH method. (**A**) Oleanolic acid, (**B**) ursolic acid, (**C**) lupeol, (**D**) octacosanol, (**E**) hexacosanal, and (**F**) γ-sitosterol (C-cyan, H-white, O-red, N-blue, S-yellow, δg^inter^ isosurface value = 0.005 a.u.). (**G**) A common interpretation of the coloring method of mapped function sign(λ_2_)ρ in IGM and IGMH maps.

In addition to forming hydrogen bonds, the weak intermolecular interaction force was visually simulated using the primary function 20 neutron function 11 in Multiwfn3.8 and optimized with VMD. The results are shown in Figure 7. IGMH analysis provides a graphical representation of intermolecular interaction regions [44]. The typical interpretations of the colouring method of mapped function sign(λ_2_)ρ in IGM and IGMH maps are shown in Figure 7G. The blue area represents the strong attraction dominated by electrostatics, corresponding to the part where hydrogen bonds can be formed in molecular docking. The green area represents the weak attraction mainly caused by the van der Waals force, and the red area corresponds to the steric hindrance effect [43]. In the IGMH approach, where ρ is the electron density, λ_2_ is the second-largest eigenvalue of the electron density Hessian matrix, sign() means to take a sign, projects the sign(λ_2_)ρ function on the isosurfaces of δg, δg^inter^, and δg^intra^ through distinct colours to display the interaction type and strength. The δg function represents the intermolecular difference between electron density gradients with and without interference [45,46]. It can be seen from the results that there are some areas where the edge of the blue isosurface is surrounded by the green isosurface, indicating that there are hydrogen bonds in these areas, which is consistent with the molecular docking results. At the same time, most of the rest are green isosurfaces, indicating that the interaction force between pesticide molecules and these waxy components in the region is dominated by the van der Waals force.

**Figure 8:**
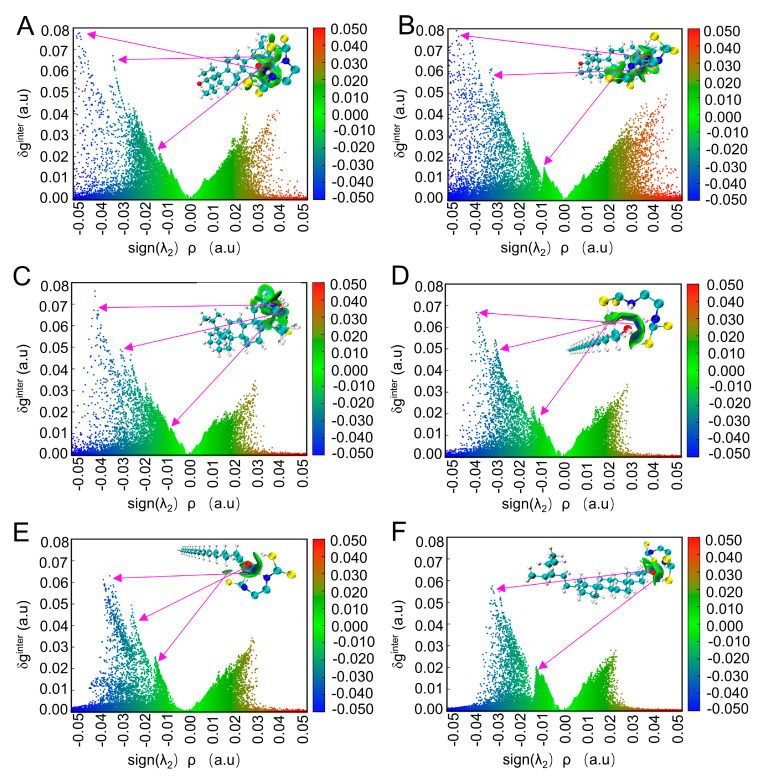
The scatter plot of δginter on sign(λ2)ρ and the projection of sign(λ2)ρ on the isosurface, the value of δginter is 0.005(a.u.). (**A**) Oleanolic acid, (**B**) ursolic acid, (**C**) lupeol, (**D**) octacosanol, (**E**) hexacosanal, and (**F**) γ−sitosterol.

The steric hindrance between molecules cannot be seen in the 3D isosurface map alone, so in the post-processing menu of the IGMH analysis, select the “−1//Draw scatter plot” option and then select the corresponding sub-options to draw a scatter plot between sign(λ_2_)ρ and different forms of δg to examine the interactions in the system. Here we mainly discuss the interaction between sign(λ_2_)ρ and δg^inter^. For clarity, the correspondence between the peak of δg^inter^ in the isosurface plot and the peak in the scatter plot is indicated by arrows, as shown in Figure 8. The figure also shows that interaction regions with larger ρ generally have more significant ordinate δg^inter^ maxima in that region. We see dense points in the area where sign(λ_2_)ρ is significantly greater than 0 in the figure, so it can be said that there is a steric hindrance in the system [47]. For example, in Figure 8A, there is a peak at the position of sign(λ_2_)ρ at about −0.04. Since the electron density at this position is not very large but not very close to 0, many blue points near the horizontal axis indicate its existence. Therefore, it can be preliminarily judged that the strong attraction should be the hydrogen bond between the mancozeb molecule and the oleanolic acid molecule; the scatter plot with sign(λ_2_)ρ around −0.01~0.01 is green, indicating that this area corresponds to the intermolecular force of is the van der Waals forces; when the sign(λ_2_)ρ is more significant than 0.02, the colour of the scatter plot changes from olive green to red, indicating that there is a steric hindrance in this region [46]. According to this analysis method, the van der Waals force is the primary interaction between the six wax constituent molecules and the mancozeb molecules. At the same time, hydrogen bonding and steric hindrance effects coexist.

### 2.4. Comparison of Mancozeb Content in Grapes

**Figure 9:**
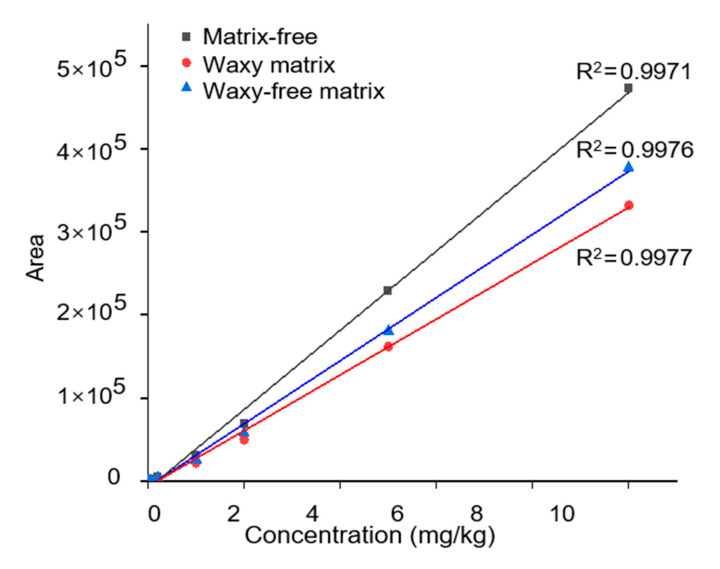
Standard curves of three mancozebs.

Method linear: The standard sample solution was created by derivatizing 1 mL of the mancozeb common solution in a 50 mL centrifuge tube. Nitrogen was used to blast a 1 mL standard sample until nearly dry. 1 mL of the two derived grape blank solutions was added for each matrix standard sample solution. Under the predetermined chromatographic conditions, the samples were injected, and the concentration of mancozeb was used as the abscissa. The area of the associated peak was used as the ordinate. Mass spectrum information of mancozeb is provided in Figure S2. A standard curve for matrix-free, a standard curve for waxy matrix, and a standard curve for wax-free matrix were prepared, as shown in Figure 9. Matrix effects refer to the fact that the co-eluted substances change the ionization efficiency of the components to be tested during chromatographic separation, resulting in the inhibition or enhancement of the signal [48]. Electrospray ionization efficiency is easily affected by the sample matrix. When the influence of the matrix effect is significant, the sensitivity of the method will be reduced, and the accuracy of the way will be affected. The addition of grape matrix showed a negative matrix effect on pesticide detection, and the presence of wax was one of the factors affecting the magnitude of the negative matrix effect. Therefore, the matrix-matched standard solution was used for calibration to eliminate the influence of matrix effects. When using the standard curve to quantify pesticide residues, whether the sample contains wax should be calculated according to different standard angles to ensure the accuracy of the results.

There have been relevant studies on the matrix effect of fruit wax layer components in detecting pesticides, such as oleanolic acid and ursolic acid in apples, which are related to detecting nine pesticides in apples by GC-MS [44]. There was a significant correlation with adverse matrix effects. The likely reason is that during sample injection, specific constituent molecules in the waxy layer can accumulate in the inlet liner and create active sites capable of reacting with the pesticide, thereby reducing the amount of pesticide reaching the mass detector. The propensity of each insecticide to interact with active molecules in the waxy layer may lead to matrix effects of various strengths [43]. A similar situation may exist in the detection of HPLC-MS.

Differences in mancozeb residues in two grape samples: Soaking in chloroform can remove the waxy layer on the surface of grapes. In contrast, mancozeb is insoluble in water (6.2 mg/L at 20 °C and K_OW_ = 21.38) and most organic solvents [26]. A comparison of mancozeb content in grapes before and after dewaxing can indirectly show the interaction between the wax layer and mancozeb.

The blank piece was spiked and recovered with pesticide standard solution. The recovery rate (as shown in Table S4) of the sample with the additional amount of 1, 2, and 10 mg/kg were 93.5~105%, 89.2~105%, and 90.2~106% (*n* = 6), and the relative standard deviations (RSD) were 4.98%, 7.00%, and 7.08%, which met the requirements. Table S5 showed that the residue of mancozeb in the grapes (K, 5.97 ± 0.47 mg/kg) after soaking with pesticides was higher than that in the grapes after the wax layer was removed (Y, 1.12 ± 0.09 mg/kg), indicating that mancozeb was present with the surface wax of the grapes. The data of the two groups of samples were tested by the independent sample *t*-test, which satisfied the homogeneity of variance within the group, and the significance between the groups was *p* < 0.05. It can be considered that there is a significant difference between the two groups of grape samples. It can be used as evidence for the speculation that there is an interaction between the wax component and the pesticide molecule through hydrogen bonding, van der Waals force, and other interaction forces, so that the mancozeb, which is insoluble in chloroform, is removed from the grape surface along with the waxy layer.

## 3. Materials and Methods

### 3.1. Grape Materials, Chemicals, and Standards

Mature *Vitis vinifera ‘Summer Black’* berries were hand-harvested from a local organic vineyard in Honghe, Yunnan Province, China. Berries of each bunch were separated from stems using scissors. Berries without rot or physical damage were selected for further experiments. The average weight per grape berry was about 10 ± 0.5 g.

Mancozeb standard (purity 70.0%) was purchased from Dr. Ehrenstorfer GmbH Co., Ltd. (Augsburg, Germany). Primary secondary amine sorbent (PSA) was obtained from Agela Technologies Co., Ltd. (Tianjin, China). L-cysteine hydrochloride (purity ≥ 98%) was acquired from J&K Scientific Co., Ltd. (Shanghai, China). Ethylenediaminetetraacetic acid disodium salt (EDTA disodium salt, purity ≥ 99%) was obtained from Innochem Technology Co., Ltd. (Beijing, China). The ultrapure water was used in the experiment, and it was prepared using a Milli-Q water system (Millipore, Billerica, MA, USA). The rest of the solvents and reagents were of analytical grade.

### 3.2. Electron Microscopy of Grape Peel

We referenced Casado et al.’s method [49] for sample preparation and made improvements. At the equator of the grapefruit, we used a blade to trim the waxy peel to about 2 mm × 2 mm × 1 mm, and fixed the peel in 2.5% glutaraldehyde. The samples were dried with a critical point dryer (Autosamdri-815A, Tousimis, Rockville, MD, USA) and attached by a conductive tape to a sampling table. A gold particle was coated with Hitachi MC1000 and examined with a Hitachi SU8020 field emission scanning electron microscope.

### 3.3. Wax Content Determination

The grapes were rinsed and then dried. Scissors were used to separate the grapes from the fruit stems, leaving behind stems that were about 2 mm in diameter. The grapes in the experimental group received CHCl_3_ treatment to dissolve the epidermal wax layer, while those in the control group were left untreated. The grapes in the two groups were immersed in a standard solution of 10 mg/L mancozeb produced in ultrapure water for 3 h before being removed and allowed to dry naturally.

The analysis method was improved according to the way mentioned [50]. The beaker was washed, dried, and weighed after cooling, and the beaker weight was recorded as *m*_1_ (g). While the processed grapes were put in the beaker, the grape weight was noted as *M* (g). Next, the CHCl_3_ was poured by a material–liquid ratio of 1:2.5 (g: mL), and the grapes were extracted using ultrasonic treatment with a power of 250 W for the 30 s at 20 °C. The above operation was repeated once, and the remaining liquid was combined and placed in a fume hood to air dry overnight. The weight of the beaker after air drying was weighed and recorded as *m*_2_ (g). The wax content was calculated according to the following Equation (1):

(1) $$\mathrm{Wax}\;\mathrm{content}=\frac{\left(m_{2}-m_{1}\right)}{M}\times 10^{6}\;(\mathrm{mg}/\mathrm{kg})$$

### 3.4. Wax Analysis

A solution containing 2 mL of CHCl_3_ and 20 mg of wax was vortexed for 1 min to dissolve the wax and was then passed through a 0.22 μm membrane. The filtrate was identified for aliphatic substances detection by GC-MS. The hydroxyl-containing compounds in grapevine wax were derivatized by the method mentioned by Arand et al. to convert them to the corresponding trimethylsilyl derivatives [7].

Wax analysis was performed on a GC-MS instrument with electron ionization and TOF detector (Pegasus BT, Laboratory Equipment Corporation, Joseph, MI, USA). Separation was carried out on a DB-5MS analytical fused silica capillary column (30 m × 0.25 mm i.d. × 0.25 μm film thickness, Agilent Technologies, Santa Clara, CA, USA). The injection volume was 2 μL. The inlet, MS transfer line, and ion source temperature were set at 250, 300, and 230 °C, respectively. The initial temperature was 50 °C for 0.5 min, and was ramped at 15 °C/min to 280 °C for 30 min. The electron energy was 70 eV. The flow rate was 1.0 mL/min of He (99.999%). The qualitative report of the mass spectrum of grape wax was preliminarily determined by being compared with the standard substance library of the instrument, and then the manual screening was carried out.

### 3.5. Simulation Analysis of the Combination of Pesticides and Waxes

Mancozeb (CID: 13307026) as a guest molecule and host molecules oleanolic acid (CID: 10494), ursolic acid (CID: 64945), lupeol (CID: 259846), octacosanol (CID: 68406), hexadecanol (CID: 3084462), and γ-sitosterol (CID: 457801) were obtained from the National Centre for Biotechnology Information (https://pubchem.ncbi.nlm.nih.gov/substance accessed on 5 January 2023) in sdf. format. All of these structures are shown in Figure S1. MM2-optimized structures were performed by ChemBio3D Ultra 20.0 (CambridgeSoft Waltham, MA, USA), and the optimized host–guest molecular systems were used for the following molecular docking calculations.

Molecular docking calculations were performed using the AutoDock 4.2 (http://autodock.scripps.edu/ accessed on 15 January 2023) software package. Non-polar hydrogen atoms from acceptor and guest molecules appeared, with added Gasteiger charges. The prediction of bound conformation by free energy was based on empirical force fields and Lamarck Genetic Algorithm (LGA) [51]. The number of genetic algorithms was 100, and other docking parameters were set to default values. The docking log file (dlg.) was analyzed by the AutoDock Tool (version 1.5.6). The binding mode with the lowest free energy was selected as the primary compulsory mode.

Multiwfn 3.8 and Visual Molecular Dynamics (VMD) [52] were used to visualize the weak intermolecular interactions of AutoDock results, the primary molecular structure visualization software in this study.

### 3.6. Detection of Pesticide Residues in Grapes

Two kinds of grape samples were prepared for the experiment.

The original grapes were submerged for 3 h in a standard solution of 10 mg/L mancozeb made with ultrapure water before being removed and were allowed to dry naturally. K should stand in for this group.

Remove the surface wax layer from the dried grapes in 1. Y is used to represent this group.

Detection of Mancozeb: The analysis method was modified from that described by López-Fernández et al. by changing the centrifugal condition and dispersing purifying agent [53]. Under the need to add antioxidant L-cysteine, mancozeb was substituted with EDTA disodium salt to produce sodium substitution, which then underwent methylation with dimethyl sulfate to obtain methyl ethylidene-1,2-dithiocarbamate (EBDC-dimethyl), a methylation derivative. The samples were cleaned up by the QuEChERS procedure and detected by the HPLC-MS method. The ion pair *m*/*z* 241/193 was selected as the quantitative ion pair.

Analysis of samples was carried out with an AB Sciex QTRAP5500 HPLC-MS system (Applied Biosystems Inc., Carlsbad, CA, USA) equipped with triple quadrupole mass spectrometry (Q-TRAP-MS) with electrospray ionization (ESI). The analytical column was an Agilent EC-C18 column (200.0 mm × 100 mm, 2.7 μm). The gradient elution mobile phase consisted of acetonitrile (eluent A) and 0.1% formic acid solution (eluent B). The optimized linear gradient system was as follows: 0–0.5 min, 5% A; 0.5–4 min to 95% A; 4–6 min, 95% A; 6–6.1 min to 5% A; 6.1–8 min, 5% A. The injection volume was 2 μL and the flow rate was 300 μL/min. Analytes were detected using ESI interface in positive-ion mode (temperature 325 °C), nebulizer pressure 40 psi, drying gas N_2_: 9 L/min, capillary voltage: +4000 V. Instrument, control, and data integration were performed using Analyst^®^ Software Version 1.6.2.

### 3.7. Data Analysis and Processing

We used Origin 2022b software to draw the graph; the result is expressed as mean ± standard deviation from three repeated tests. IBM SPSS 25.0 software was used to conduct an independent sample T-test and One-Way ANOVA significance analysis on the data for the significance of differences. The results of *p* < 0.05 were regarded as statistically significant.

## 4. Conclusions

This work investigated the interaction of pesticide molecules with the waxy layer on the grape surface and their effects on pesticide residues in grapes. The exposure to mancozeb altered the components of the waxy layer of grapes, including triterpenoids (e.g., oleanolic acid, ursolic acid, and lupeol), sterol (e.g., γ-sitosterol), long-chain alkyl alcohols (e.g., octacosanol), and long-chain alkyl aldehydes (e.g., hexacosanal). Molecular simulation studies revealed that the six waxy components (oleanolic acid, ursolic acid, lupeol, octacosanol, hexacosanal, and γ-sitosterol) interacted with mancozeb via hydrogen bonding and van der Waals forces. The corresponding binding energies were −5.96, −5.87, −3.30, −3.67, −3.82, and −2.22 kcal/mol, respectively. This intermolecular interaction would make mancozeb easier to deposit on the surface of grapes. We also determined the residues of mancozeb in grapes by the QuEChERS-HPLC-MS method. Significant differences in the content of mancozeb in grapes were observed before and after dewaxing, further suggesting that the intermolecular interaction resulted in more pesticide residues in grapes.

## Data Availability

Data are contained within the article and Supplementary Materials.

## Supplementary Materials

The following supporting information can be downloaded at: https://www.mdpi.com/article/10.3390/ijms24097705/s1.
