# Supplementary material for: Interaction between Six Waxy Components in Summer Black Grapes (Vitis vinifera) and Mancozeb and Its Effect on the Residue of Mancozeb
Source: Int J Mol Sci. 2023 Apr 22;24(9):7705. doi: 10.3390/ijms24097705 (PMC10178566; doi:10.3390/ijms24097705)
Supplement: Supplementary file 1 [file ijms-24-07705-s001.zip › ijms-2327348-supplementary.pdf]

Table S1. Statistical table of wax extraction weight of fresh grapes and pesticide grapes (unit: mg/kg)

number	fresh grapes	pesticide grapes
1	268	334
2	264	306
3	322	335
4	348	427
5	336	336
6	320	302
7	337	251
8	310	340
9	299	328
10	385	340
Average	319±36.3	329±43.8
RSD	11.5%	13.9%
P	> 0.05	

Table S2 Some information about a part of components

mcromolecule	formula	CAS	retention time (min)
[ethylenebis-(dithiocarbamato)]-manganese	$C_4H_6MnN_2S_4$	301-03-1	8.9
oleanolic acid	$C_{30}H_{48}O_3$	508-02-1	52.9[a]
ursolic acid	$C_{30}H_{48}O_3$	77-52-1	53.5[a]
lupeol	$C_{30}H_{50}O$	545-47-1	20.5
octacosanol	$C_{28}H_{58}O$	557-61-9	22.4
hexacosanal	$C_{26}H_{52}O$	26627-85-0	18.9
γ-sitosterol	$C_{29}H_{50}O$	83-47-6	17.1
undecane	$C_{11}H_{24}$	1120-21-4	6.03
2,2'-methylenebis(4-methyl-6-tert-butylphenol)	$C_{23}H_{32}O_2$	119-47-1	15.9
ethyl tetracosanoate	$C_{26}H_{52}O_2$	24634-95-5	18.5
heptadecanoic acid ethyl ester	$C_{19}H_{38}O_2$	14010-23-2	20.7

[a] indicates that the substance needs to be transformed into their corresponding trimethylsilyl derivatives by incubating in bis-N, O-(trimethylsilyl)trifluoroacetamide (BSTFA) so that it can be detected by GC-MS

Table S3 The docking results of mancozeb with six molecules at 298.15 K

macromolecule	number of run	lowest binding energy (kcal/mol)	estimated Inhibition constant, Ki (mM, 298.15K)	integral of δg^{inter}(a.u.)	sum of all atomic pair δg indices
oleanolic acid	46	-5.96	0.04	0.803	3.15
ursolic acid	56	-5.87	0.05	0.858	3.32
lupeol	89	-3.30	3.83	0.646	2.53
octacosanol	93	-3.67	2.04	0.487	1.82
hexacosanal	39	-3.82	1.58	0.419	1.32
γ-sitosterol	81	-2.22	23.6	0.719	2.74

Table S4 Recoveries of mancozeb at 1.0, 2.0, and 10.0 mg/kg

concentration (mg/kg)	recovery (%)						average (%)	RSD (%)
	n_1	n_2	n_3	n_4	n_5	n_6		
1.0	96.4	93.5	105	95.6	102.6	93.5	97.8	4.98
2.0	105	94.6	99.1	104	90.6	89.2	97.1	7.00
10.0	95.6	90.2	104	97.0	108.8	106	100.2	7.08

Table S5. Statistical table of mancozeb residues in two groups of grapes

name	area	concentration (mg/kg)	average (mg/kg)
K1	2.37E+05	6.47	
K2	2.16E+05	5.93	5.97±0.47
K3	2.00E+05	5.52	
Y1	2.86E+04	1.04	
Y2	3.01E+04	1.09	1.12±0.09
Y3	3.45E+04	1.22	

K ： mancozeb content in grapes before dewaxing

Y： mancozeb content in grapes after dewaxing

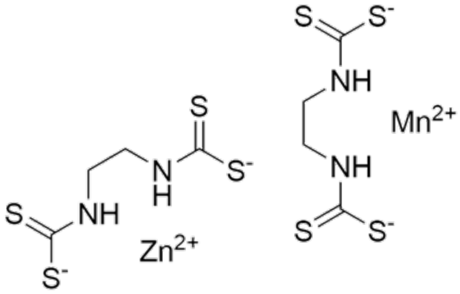

mancozeb

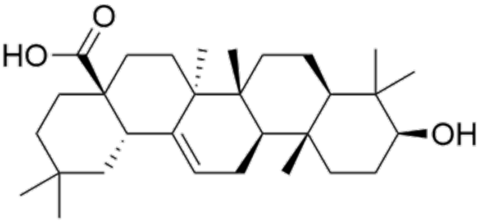

oleanolic acid

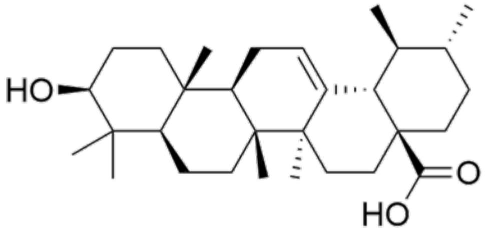

ursolic acid

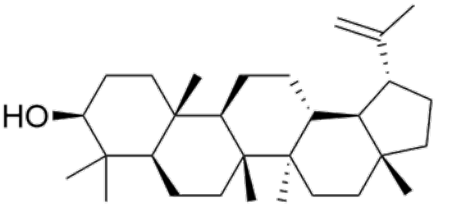

lupeol

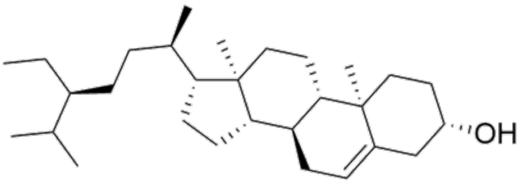

γ-sitosterol

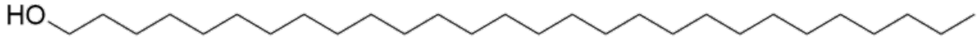

octacosanol

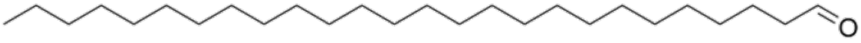

hexadecanol

Figure S1 The structures of oleanolic acid, ursolic acid, lupeol, octacosanol, hexacosanal, γ-sitosterol and mancozeb

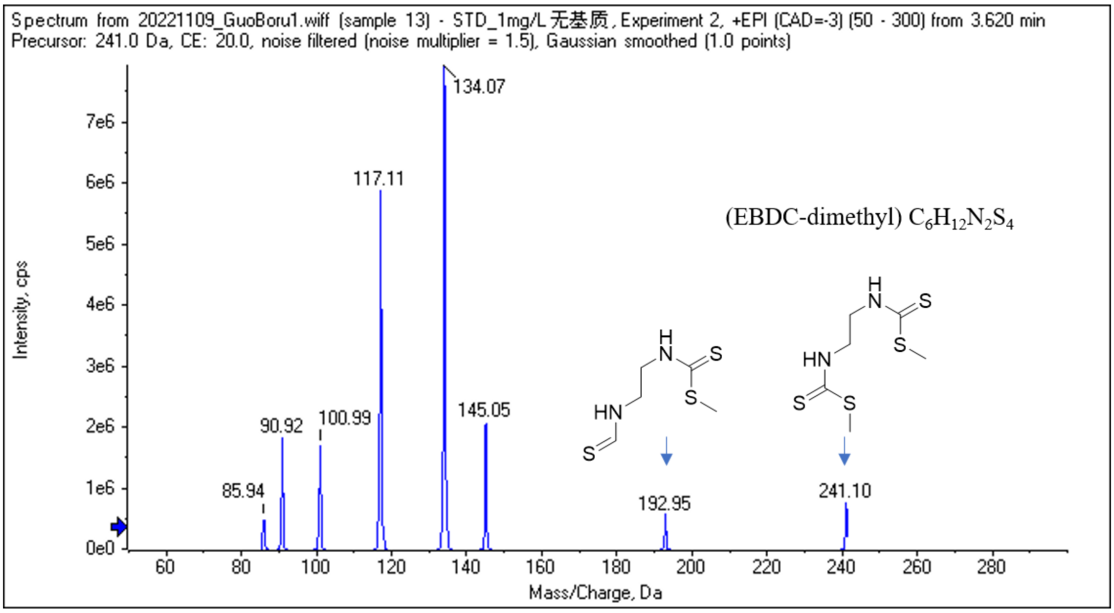

Figure S2 The mass spectrogram of mancozeb.
